# Resource Utilization of Limestone Powder and Steel Slag Powder in Cement-Based Materials at Extremely Low Water/Binder Ratio

**DOI:** 10.3390/ma19173662

**Published:** 2026-08-28

**Authors:** Yuchen Han, Yuchen Li, Hongyi Zhang, Qihan Liu, Fanghui Han

**Affiliations:** 1 Department of Civil Engineering, School of Future Cities, University of Science and Technology Beijing, Beijing 100083, China; m202320159@xs.ustb.edu.cn (Y.H.); m202220145@xs.ustb.edu.cn (Y.L.); m202420205@xs.ustb.edu.cn (H.Z.); m202420168@xs.ustb.edu.cn (Q.L.); 2 Beijing Key Laboratory for Urban Safety and Collective Intelligence, University of Science and Technology Beijing, Beijing 100083, China; 3 Hebei Key Laboratory of Collapse Prevention, University of Science and Technology Beijing, Beijing 100083, China

**Keywords:** limestone powder, steel slag powder, curing conditions, extremely low w/b ratio, compressive strength, hydration

## Abstract

The large-scale resource utilization of limestone powder generated from mining operations and steel slag waste discharged from the metallurgical industry is of vital importance. The effects of limestone powder or steel slag powder on the properties of cement-based materials at an extremely low water/binder (w/b) ratio of 0.16 under standard curing and early high-temperature curing at 60 °C for 3 d were comparatively studied through measuring the compressive strength, hydration degree and microstructure. The results show that limestone powder promotes the early strength development of concrete at an extremely low w/b ratio better than steel slag powder does under standard curing conditions, but steel slag powder promotes the late strength growth better. For concrete containing 10% and 20% steel slag powder, the compressive strength increased by 20.4 and 33.7 MPa, respectively, from 3 to 7 d, and the difference in their compressive strengths was only 0.4 MPa at 90 d. The limestone powder promotes strength more significantly under early high-temperature curing conditions; however, when the dosage reaches 30%, the long-term strength of the concrete containing steel slag powder is marginally greater compared to the concrete containing limestone powder. At 90 d under early high-temperature curing, the chemically bound water contents of the pastes containing limestone powder ranged from 9.05% to 10.53%, compared with 10.64% to 11.85% for the pastes containing steel slag powder. The diffraction peak intensity of ettringite is stronger in the paste with limestone powder due to the stabilizing effect of limestone powder on ettringite. The diffraction peak intensity of Ca(OH)_2_ is stronger in the paste with steel slag powder, which exhibits a higher hydration degree and generates more hydration products. The pore structure of the sample containing steel slag powder improves, and the microstructure becomes denser at high temperatures.

## 1. Introduction

Globally, carbon dioxide (CO_2_) emissions from the concrete industry account for approximately 26% of total industrial emissions [1], and approximately 88% of carbon emissions originate from cement [2]. From the perspective of low-carbon sustainable development, the consumption of cement in concrete production must be reduced [3]. At present, the use of industrial by-products instead of cement is an ideal choice [4,5,6]. In cementitious material systems, industrial by-products are further processed into supplementary cementitious materials (SCMs) to replace cement [7,8]. SCMs are characterized by low carbon dioxide emissions and the ability to conserve natural resources [9]. Many kinds of SCMs have been developed for the production of concrete, such as silica fume, ground granulated blast-furnace slag (GGBFS), steel slag powder, and limestone powder [10,11,12]. Currently, with the development of modernization in China, the demand for cement-based materials at an extremely low w/b ratio has greatly increased due to its excellent properties, such as ultra-high-performance concrete (UHPC). An important way to achieve green and sustainable development is to prepare UHPC by using high-content SCMs instead of cement [13]. As a high-quality mineral admixture, GGBFS has also become a scarce resource. Therefore, the use of steel slag powder, limestone powder and other relatively low-activity admixtures with GGBFS to prepare UHPC has become a research hotspot.

Steel slag powder is an industrial by-product of the steelmaking process and can undergo hydration reactions [14,15]. The hydration products of steel slag powder are almost the same as those of cement, they influence each other’s hydration by altering the hydration environment [16]. Steel slag powder slows down the early hydration of cement. Adding steel slag powder raised the Ca^2+^ concentration in the pore solution, which reduced CH supersaturation in the pore solution, hindering the nucleation and growth of C-S-H gel, thereby postponing early cement hydration [17]. Related studies have evaluated the performance of UHPC with steel slag powder and reported that the addition of steel slag powder improved the workability of UHPC and that the early strength of UHPC decreased with increasing dosage of steel slag powder, but the late strength was not affected [18]. The self-shrinking strain of the UHPC decreased with increasing steel slag powder content, which was primarily attributed to the low hydration activity of steel slag powder. Additionally, unhydrated particles further contributed to the inhibition of shrinkage.

The role of limestone powder in cementitious materials encompasses filling, nucleation, dilution, and chemical interactions [19,20]. The filling effect of the limestone powder refined the microstructure of the cementitious materials and reduced porosity [21]. The nucleation effect of the limestone powder provided many nucleation sites for the formation of hydration products, accelerating the hydration of C3S and increasing the amount of hydration products. The dilution effect of limestone powder reduced the exothermic peak in the early stage of hydration. The chemical effect of limestone powder promoted the emergence of the third exothermic peak of cementitious materials due to the formation of carboaluminate [20]. Yu et al. [22] partially substituted cement with GGBFS powder or limestone powder in UHPC and reported that both powders had comparable impacts on the early hydration of UHPC; however, the hydration rate of the cementitious system with GGBFS was faster at a later age, and its mechanical properties were better than those of UHPC with limestone powder. Boubekeur et al. [19] reported that the compound mineral admixture containing limestone powder and GGBFS had a good complementary effect on the mechanical properties of mortar.

Recent studies have explored different strategies for reducing the cement content of UHPC through the incorporation of SCMs. For limestone powder, current research has mainly focused on its influence on fresh-state rheology, particle packing, water-film thickness, and its synergistic use with other mineral admixtures, such as calcined clay [23,24]. For steel slag powder, recent studies have investigated its use in multi-component or optimized UHPC systems, particularly in combination with other solid-waste powders [25,26]. Other investigations have demonstrated the feasibility of developing low-cement UHPC and optimizing ternary sustainable binder systems [27,28]. However, these studies generally considered a single supplementary cementitious material or a specific hybrid binder, and a direct comparison between limestone powder and steel slag powder under identical binder compositions, replacement levels, and curing regimes remains limited. This research gap is particularly relevant at the extremely low w/b ratio. In this study, Portland cement was limited to 50% of the total binder, while SCMs constituted the remaining 50%, with the aim of evaluating the feasibility of using high-volume mineral admixtures in a cementitious system oriented toward UHPC-level performance. The low w/b ratio was adopted to limit capillary porosity and promote the formation of a dense matrix, thereby compensating for the reduction in early reactivity associated with the high replacement level. The aim of this study is to reveal the mechanism of limestone powder and steel slag powder in cement-based materials at an extremely low w/b ratio with a low clinker content through a comprehensive study of the hydration and microstructure while ensuring their mechanical properties. The distinctive contribution of this work is the direct comparison of limestone powder and steel slag powder at replacement levels of 10%, 20%, and 30% within the same binder system under both standard curing and early high-temperature curing. The impacts of limestone powder and steel slag powder on hydration and hardening properties of cement-based materials at very low w/b ratios are evaluated through mechanical properties, hydration products, pore structure, and micromorphology analysis. Finally, this study provides a theoretical basis and data support for the application of limestone powder and steel slag powder in low-clinker cement-based materials at an extremely low w/b ratio.

## 2. Experimental

### 2.1. Materials

**Figure 1:**
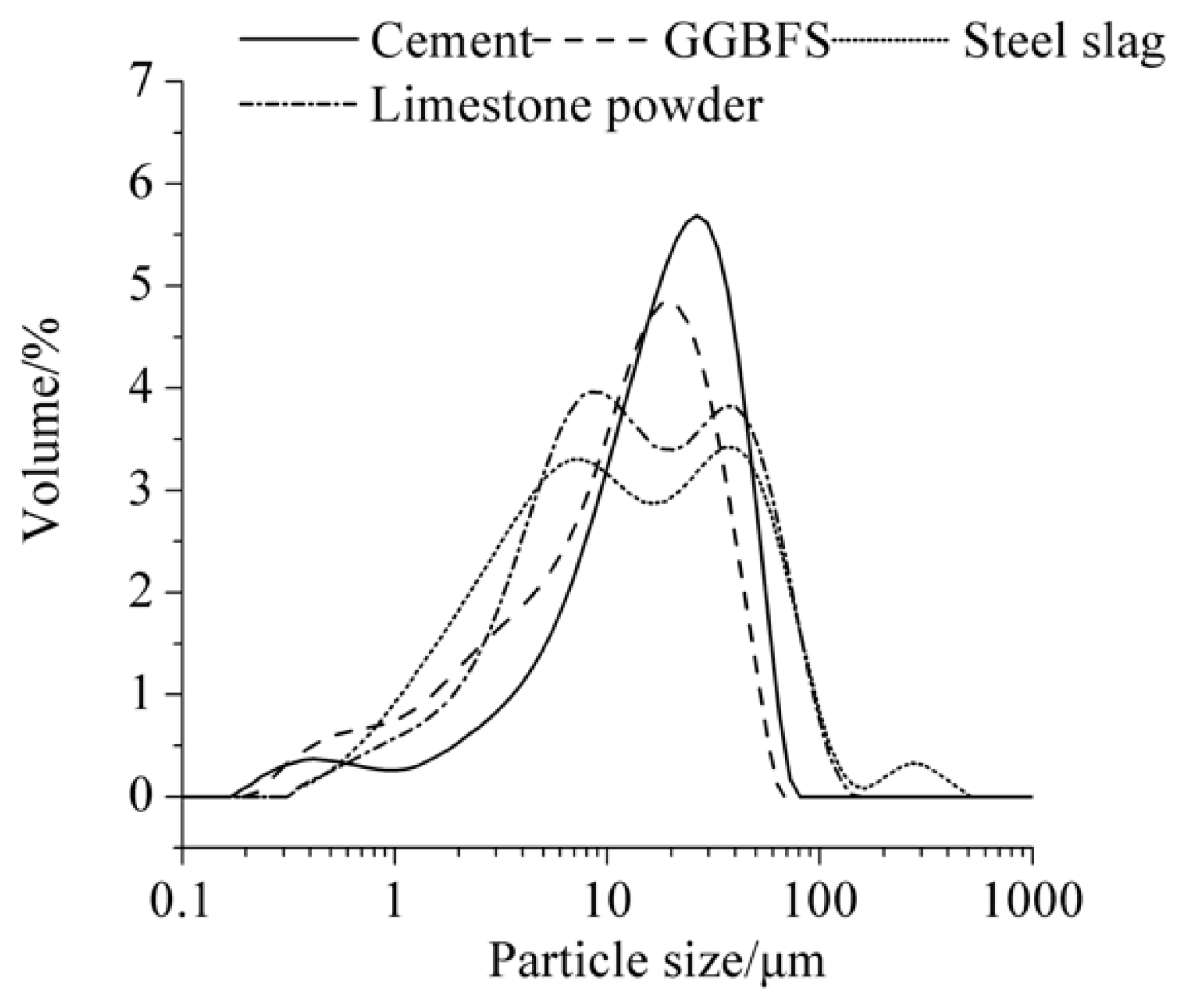
Particle size distributions of the cement, GGBFS, steel slag powder and limestone powder.

P·I 42.5 Portland cement (supplied by China Building Materials Academy, Beijing, China), GGBFS, steel slag powder and limestone powder (supplied by Shandong JinTaicheng Construction Technology Co., Ltd., Linyi, China), and silica fume (provided by China Construction Science and Technology Group Co., Ltd., Beijing, China) were used in this study. The chemical compositions of these raw materials are shown in Table 1. Figure 1 presents the particle size distributions of the four raw materials. The critical particle sizes of the cement, GGBFS, steel slag powder and limestone powder are 30 μm, 20 μm, 7 μm and 9 μm, respectively. The pore size distributions of the limestone powder and steel slag powder are similar, with pore sizes distributed mainly in the range of 8–100 μm, and their particle sizes are finer than those of Portland cement. However, several large particles ranging from 200~300 μm in size are present in the steel slag powder. A polycarboxylate-based high-range water-reducing admixture (HRWRA) provided by Jiangsu Sobute New Materials Co., Ltd. (Nanjing, China) was employed, with a content of 2.0% by mass of the total binder. The water contained in the liquid HRWRA was included in the total mixing-water content when calculating the w/b ratio.

### 2.2. Mix Proportions

The mix proportions of the concrete and paste with w/b ratios of 0.16 are displayed in Table 2 and Table 3, respectively. The mixture design was developed with reference to previous research demonstrating the feasibility of incorporating up to 50% mineral admixtures into eco-friendly UHPC [29]. The main purpose of the mixture design was to evaluate the feasibility of using high-volume mineral admixtures in UHPC while reducing the clinker content. Therefore, the Portland cement content was fixed at 50% by mass of the total binder, while the total content of mineral admixtures was maintained at 50%. Silica fume was fixed at 10% in all mixtures to eliminate the influence of variations in its content. Limestone powder or steel slag powder was incorporated at 10%, 20%, and 30% by mass of the total binder, and the GGBFS content was correspondingly reduced from 30% to 10%. The three replacement levels were selected to systematically evaluate the feasibility and performance of these materials at low, moderate, and relatively high dosages in an extremely low-w/b cementitious system. A w/b ratio of 0.16 was selected to represent an extremely low-w/b cementitious system.

### 2.3. Test Methods

Two curing methods were adopted in this experiment: standard curing condition (SCC) (20 ± 1 °C and ≥95% relative humidity (RH)) and early high-temperature curing condition (HTCC) (60 °C for 3 days and ≥95% relative humidity (RH)) where the material was then put into a standard curing room for maintenance until the specified age was reached.

Concrete at an extremely low w/b ratio was prepared according to Table 2. The compressive strength of the concrete was measured at specified ages of 3, 7, 28 and 90 days under different curing conditions.

The pastes were prepared according to Table 3. The chemically bound water contents (Wn) of the hardened pastes were measured at 3, 7, 28 and 90 days under different curing conditions. The chemically bound water content in the hardened paste was quantified using a high-temperature burning method.

The hydration products of the hardened paste with steel slag powder or limestone powder were measured via a TTR III X-ray diffractometer, with a 2θ scale scanning range of 5–65° at a rate of 5°/min.

The pore size distribution of the hardened paste was determined when the paste was cured for 28 d. The pore characteristics of the hardened paste, including porosity, pore size distribution and cumulative pore volume, were determined via an AUTOPORE II 9220 mercury injection porosimeter with a maximum mercury inlet pressure of 300 MPa.

The microscopic morphology of the hardened pastes was observed when the pastes were cured for 28 d. The samples were dried and sprayed with gold. The backscattered electron images were observed under high-vacuum conditions via a FEI Quanta 200 FEG scanning electron microscope. The accelerating voltage was 25 kV. Different brightness values were observed in the BSE images because of the different average atomic numbers of the substances.

## 3. Results and Discussion

### 3.1. Compressive Strength

**Figure 2:**
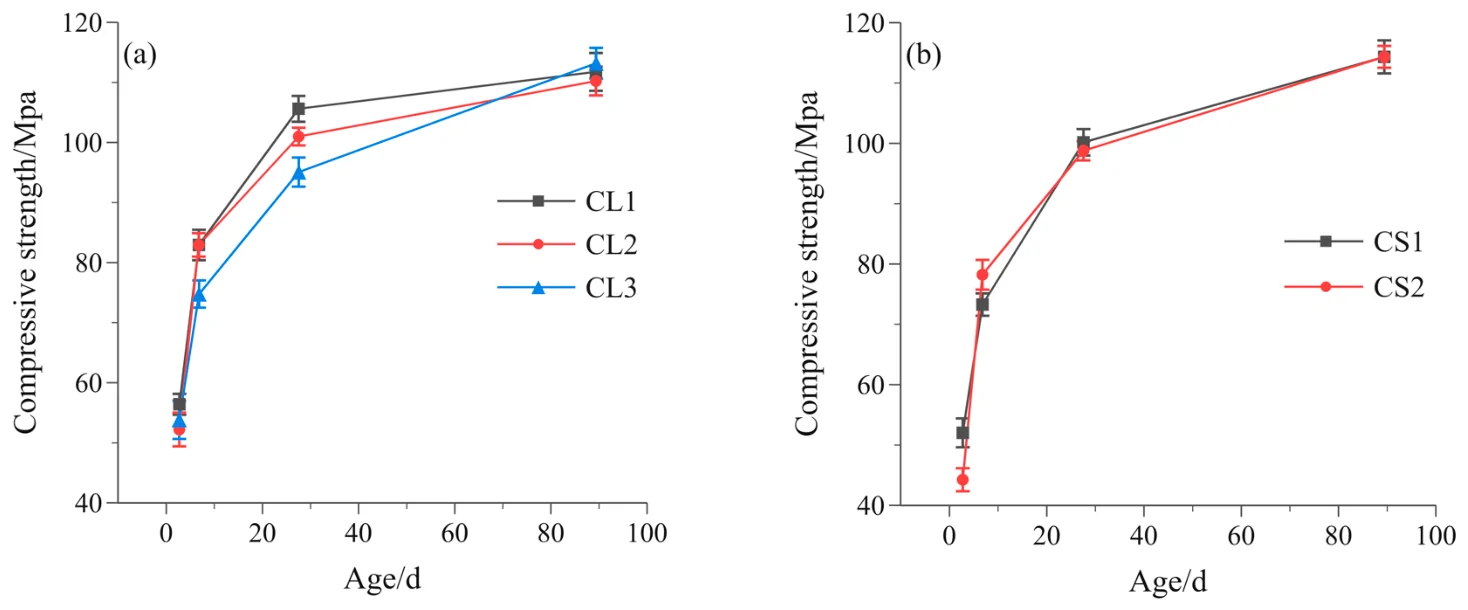
Compressive strength of concrete at extremely low w/b ratio with steel slag powder or limestone powder under SCC. (**a**) Limestone powder and (**b**) steel slag powder.

Figure 2 shows the compressive strength of concrete with limestone powder or steel slag powder under SCC. The early compressive strength of sample C_S_3 is too low. Thus, its compressive strength curve is not plotted in the figure. As shown in Figure 2a, the early compressive strength of concrete at a very low w/b ratio decreases with increasing the quantity of limestone powder content. Although limestone powder has limited intrinsic reactivity, its fine particles provide additional heterogeneous nucleation sites for the precipitation of hydration products. Its dilution effect also increases the water and space available per unit of reactive binder. Han et al. [30] reported that ultrafine limestone powder increased the early hydration rate and cumulative hydration heat of Portland cement paste, confirming that this acceleration is closely related to particle fineness and heterogeneous nucleation. However, in this system, these beneficial effects could not fully compensate for the reduction in reactive GGBFS as the limestone powder content increased, resulting in lower early strength. The strength growth rates of samples CL1 and CL2 did not differ after 28 d, but the strength growth rate of sample CL3 was much greater than that of the other two samples, and the compressive strength was the highest at 90 d. High limestone powder content provides an effective dilution effect, greatly accelerating the hydration of Portland cement, GGBFS, and silica fume. Additionally, the filling effect of limestone powder compacts the structure. Figure 2b shows that sample CS1 exhibits higher strength than sample CS2 within 3 days, suggesting that increased steel slag powder content hinders early compressive strength development in concrete at very low w/b ratios. Steel slag powder has a certain inhibitory effect on cement hydration in the early stage of hydration. A greater steel slag powder content results in a more obvious inhibitory effect. Steel slag increases the Ca^2+^ concentration in the pore solution, reduces the supersaturation with respect to CH, and inhibits the precipitation and growth of CH and C-S-H, thereby retarding early cement hydration [19]. Therefore, the early inhibitory effect becomes more pronounced as the steel slag powder content increases. When the dosage of steel slag powder is 30%, sample CS3 still cannot be demolded at 3 d. The compressive strengths of samples CS1 and CS2 increase by 20.4 MPa and 33.7 MPa from 3 d to 7 d, respectively. However, the growth rate of sample C2S is lower than that of sample CS1 after 7 d, and the difference in compressive strength between the two groups is only 0.4 MPa at 90 d. This finding indicates that the synergistic effect of steel slag powder and GGBFS increases with decreasing ratios of steel slag powder to GGBFS. When the quantity of limestone powder and steel slag powder is the same, at extremely low w/b ratios, concrete with limestone powder shows slightly higher early compressive strength than that with steel slag powder. Limestone powder accelerates early cement hydration through its nucleation and dilution effects, while steel slag powder hinders early hydration. After 28 days, the compressive strength of concrete with steel slag powder grows more rapidly at equivalent quantities, reaching the highest compressive strength at 90 days. The hydration degree of cementitious materials at an extremely low w/b ratio with steel slag powder increases due to the concerted reaction between steel slag powder and GGBFS at a later age. At later ages, the continued dissolution and reaction of steel slag may provide Ca-containing species and an alkaline environment, while GGBFS supplies reactive silicate and aluminate species, favoring the formation of additional C-(A)-S-H products. Thus, adding steel slag powder is favorable for the development of later-age compressive strength of concrete at an extremely low w/b ratio.

**Figure 3:**
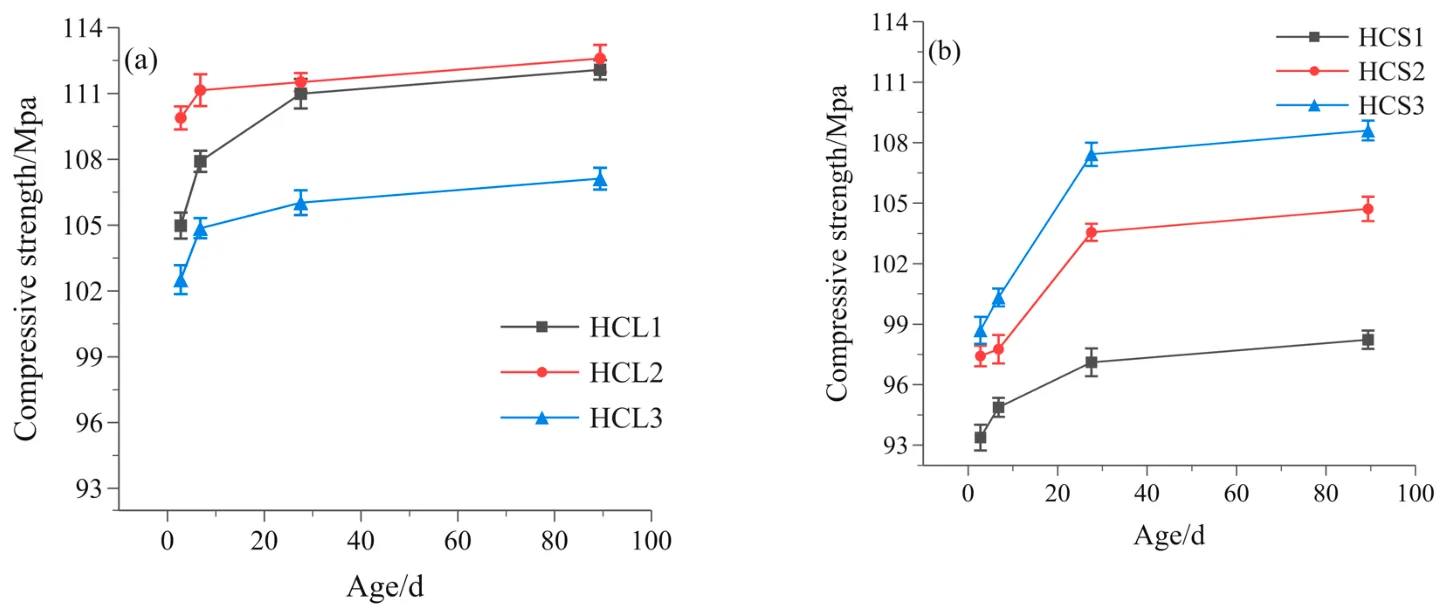
Compressive strength of concrete at extremely low w/b ratio with steel slag powder or limestone powder under HTCC. (**a**) Limestone powder and (**b**) steel slag powder.

Figure 3 shows the compressive strength of concrete with limestone powder or steel slag powder at an extremely low w/b ratio under early HTCC. As shown in Figure 3a, the compressive strength of sample HCL2 is the highest during the whole hydration age, followed by samples HCL1 and HCL3. The dispersion of highly active cementitious materials in the system increases as limestone powder content increases, which makes the distribution of hydration products more uniform. Thus, the compressive strength gradually increases. However, the thermal expansion coefficients of limestone powder and hardened paste are different under HTCC. The adhesion between the surface and the hardened paste is weak. Moreover, the total amount of highly active cementitious material in the system decreases when the content of limestone powder reaches 30%. Therefore, the compressive strength of sample HCL3 is the lowest. The strength growth rate of sample HCL1 is the highest from 3 days to 7 days, which is related to the highest content of GGBFS. Figure 3b shows that the compressive strength of concrete at an extremely low w/b ratio gradually increases with increasing steel slag powder content under HTCC. The high temperature stimulates the activity of steel slag powder, accelerates the hydration process of steel slag powder, promotes the synergistic effect of steel slag powder and GGBFS and generates more hydration products, thus increasing the compressive strength. A comparison of Figure 3a with Figure 3b reveals that the compressive strength of concrete at an extremely low w/b ratio with limestone powder is greater when the content of steel slag powder or limestone powder is 10% or 20%. However, when the quantity reaches 30%, the late compressive strength of concrete with steel slag powder is slightly greater than that of concrete with limestone powder. The strength growth rate of concrete with limestone powder under an extremely low w/b ratio is faster than that of concrete with steel slag powder before 3 d. The particle size of limestone powder is smaller than that of steel slag powder (Figure 1), and its nucleation effect is significant. The compressive strength of the sample with steel slag powder under an extremely low w/b ratio increases rapidly from 7 d to 28 d. The synergistic effect between the GGBFS reaction and steel slag powder hydration is more significant due to early HTCC, which is consistent with relevant research results [17].

By comparing Figure 2 and Figure 3, it can be seen that for the concrete with limestone powder at an extremely low w/b ratio, the compressive strength of each sample continues to grow rapidly after 28 days under SCC, whereas the strength growth slows after 28 days under HTCC. Elevated temperatures significantly enhance the strength development of concrete with steel slag powder, with a more pronounced effect at higher steel slag powder content. However, compared to SCC, the late strength of all samples is lower under HTCC. The high temperature stimulates the activity of cementitious material in the early stage, resulting in a thicker layer of hydration products on the surface of the unhydrated particles. As a result, the reaction is more difficult at the later stage of hydration. Moreover, the products generated in the system are unevenly distributed, making the late strength of the concrete samples at an extremely low w/b ratio under early high-temperature curing slightly lower.

### 3.2. Chemically Bound Water Content

**Figure 4:**
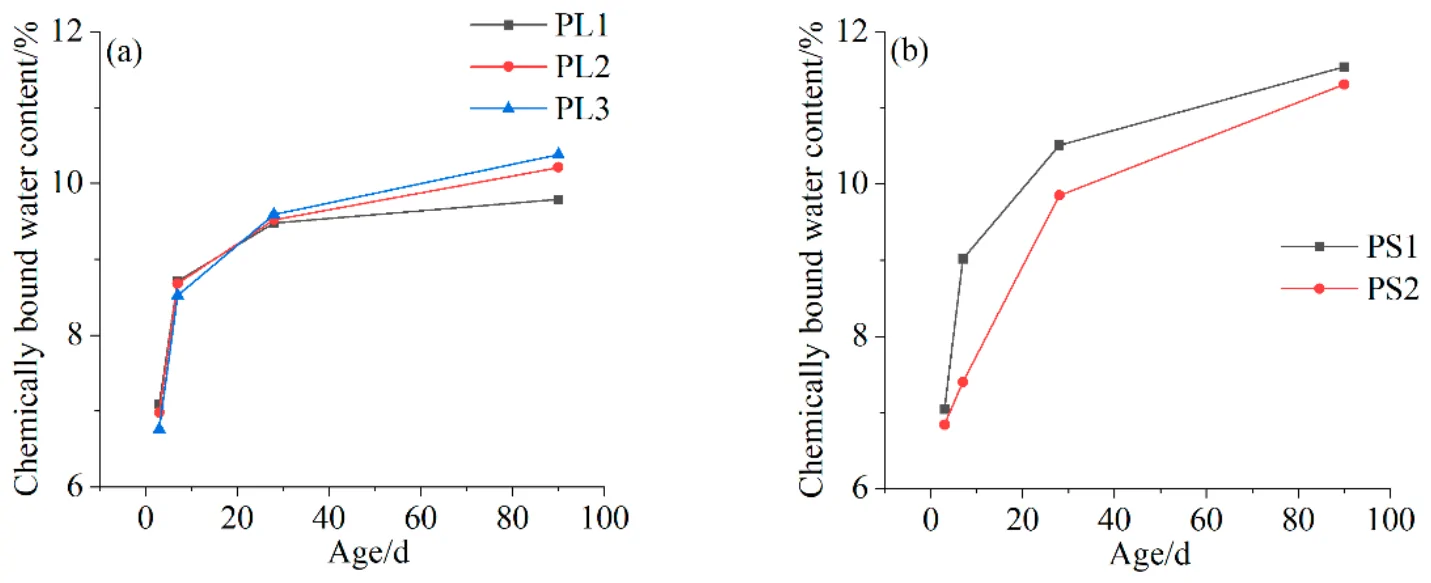
Chemically bound water content of the hardened paste at extremely low w/b ratio with steel slag powder or limestone powder under SCC. (**a**) Limestone powder and (**b**) steel slag powder.

Figure 4 shows the chemically bound water content (Wn) of the hardened paste at an extremely low w/b ratio with limestone powder or steel slag powder under SCC. Figure 4a shows that the Wn gradually decreases with increasing limestone powder content at 3 d and 7 d. Although the dilution and nucleation effects of limestone powder can increase the degree of hydration of the system, its activity is much lower than that of other cementitious materials. As a result, the Wn in the early hydration stage decreases with increasing limestone powder content. From 7 d to 28 d, the growth rate of the Wn of sample PL3 is the fastest, followed by those of samples PL2 and PL1. The Wn of samples PL1, PL2 and PL3 at 90 d are 9.79%, 10.12% and 10.38%, respectively. Owing to the dilution effect of a large amount of limestone powder, the hydration environment of sample PL3 in the later stage is improved, and the total amount of cementitious materials involved in the reaction increases, thus increasing the degree of reaction. As shown in Figure 4b, the Wn gradually decreases with increasing steel slag powder content. Compared with that of sample PS1, the growth rate of the Wn of sample PS2 decreases by approximately 18% at 3 d. Because the amount of steel slag powder in sample PS2 is greater, the reaction degree of sample PS2 is lower due to the lower activity of steel slag powder. After 7 d, the growth rate of the Wn of sample PS2 is greater than that of sample PS1. The Wn of sample PS2 is slightly lower than that of sample PS1 at 90 d. The Wn of samples PS1 and PS2 at 90 d is 11.54% and 11.31%, respectively. The hydration of steel slag powder is promoted with the progress of the pozzolanic reaction of GGBFS at a later age. Therefore, the growth rate of the Wn of sample PS2 is greater. A comparison of the data in Figure 4a,b reveals that the early reaction rate of the cementitious material with limestone powder is relatively high, indicating that limestone powder significantly promotes early hydration. However, the reaction rate of the cementitious material with steel slag powder is significantly faster at later ages. This is due to the higher activity of steel slag powder and the synergistic effect of GGBFS and steel slag powder.

**Figure 5:**
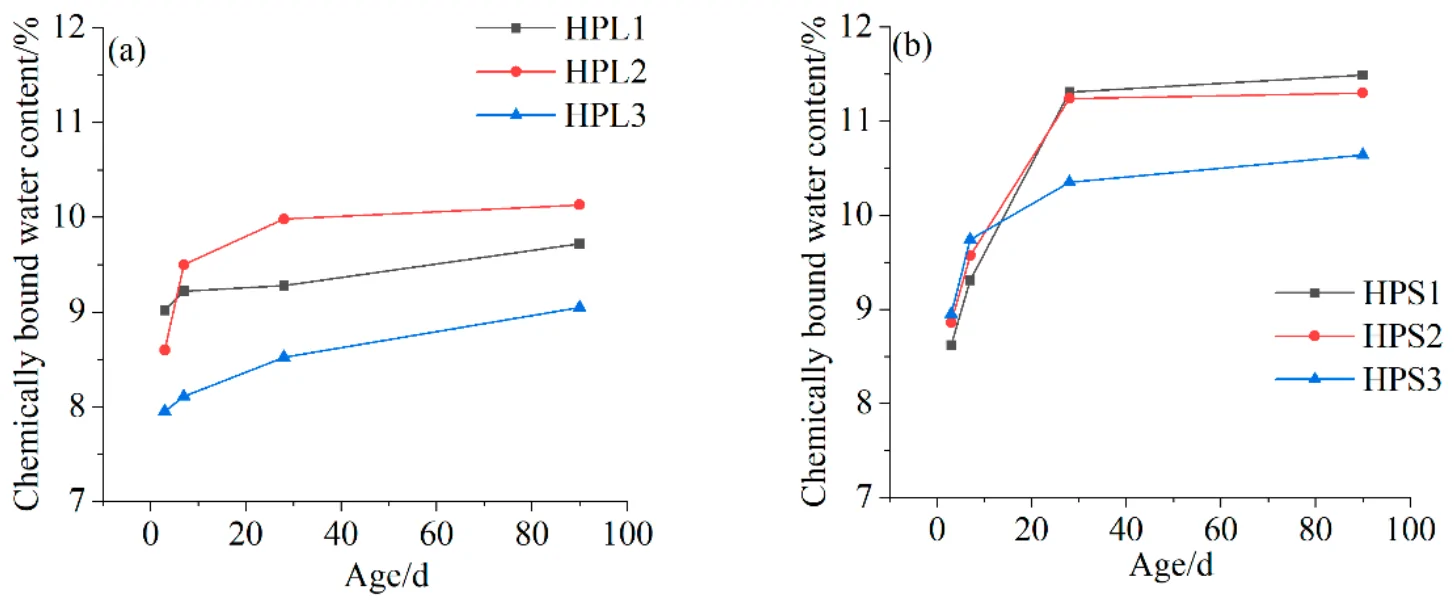
Chemically bound water content of the hardened paste at extremely low w/b ratio with steel slag powder or limestone powder under HTCC. (**a**) Limestone powder and (**b**) steel slag powder.

Figure 5 shows the Wn of hardened paste with limestone powder or steel slag powder under HTCC. Figure 5a shows that the Wn of each sample gradually increases with increasing limestone powder content at 3 d. The growth rate of the Wn of sample HPL2 from 3 d to 7 d is the highest. The results indicate that the dilution and nucleation effects of limestone powder have the most significant promoting effect on the hydration of the binder when the content of limestone powder is 20%. The Wn of sample HPL2 is the highest at 7 d and 28 d, followed by those of samples HPL1 and HPL3. The Wn of samples HPL1, HPL2 and HPL3 at 90 d are 9.81%, 10.53% and 9.05%, respectively. As shown in Figure 5b, the Wn of each sample gradually increases with increasing steel slag powder content at 3 d and 7 d. High temperatures stimulate the activity of steel slag powder. A higher content of steel slag powder leads to a more obvious promotion effect at early age. After 7 d, the growth rate of the Wn of sample HPS1 is the highest, followed by those of samples HPS2 and HPS3. The hydration products of cement and steel slag powder promote the pozzolanic reaction of GGBFS. A greater amount of GGBFS results in the generation of more hydration products. The Wn of samples HPS1, HPS2 and HPS3 at 90 d are 11.85%, 11.60% and 10.64%, respectively. A comparison of the data in Figure 5a,b reveals that, when the dosage is 10%, the Wn of hardened paste with limestone powder is greater than that of the hardened paste with steel slag powder at 3 d. When the contents are 20% and 30%, the Wn of the hardened paste with steel slag powder is much greater. The Wn of the hardened paste with steel slag powder is higher at later age. The synergistic effect of steel slag powder and GGBFS promotes the reaction of the cementitious system.

A comparison of Figure 4 with Figure 5 reveals that the Wn of the hardened paste increases rapidly under HTCC at early age but is slightly lower than that of the hardened paste under SCC at later age. High-temperature curing rapidly generates substantial hydration products. The hydration products have no time to diffuse and cover the surface of unhydrated particles, hindering the late hydration of the cementitious material and reducing the late-age Wn. For the hardened paste with limestone powder, the Wn increases slowly under early HTCC, which is the same as the compressive strength results (Figure 3), indicating that the degree of reaction of the limestone powder is extremely low. For the hardened paste with steel slag powder, because of the higher activity of steel slag powder under HTCC, the early hydration rate of steel slag powder is fast, and more hydration products are generated. The Wn of the hardened paste with steel slag powder is similar under the two curing conditions at a later age.

### 3.3. XRD

**Figure 6:**
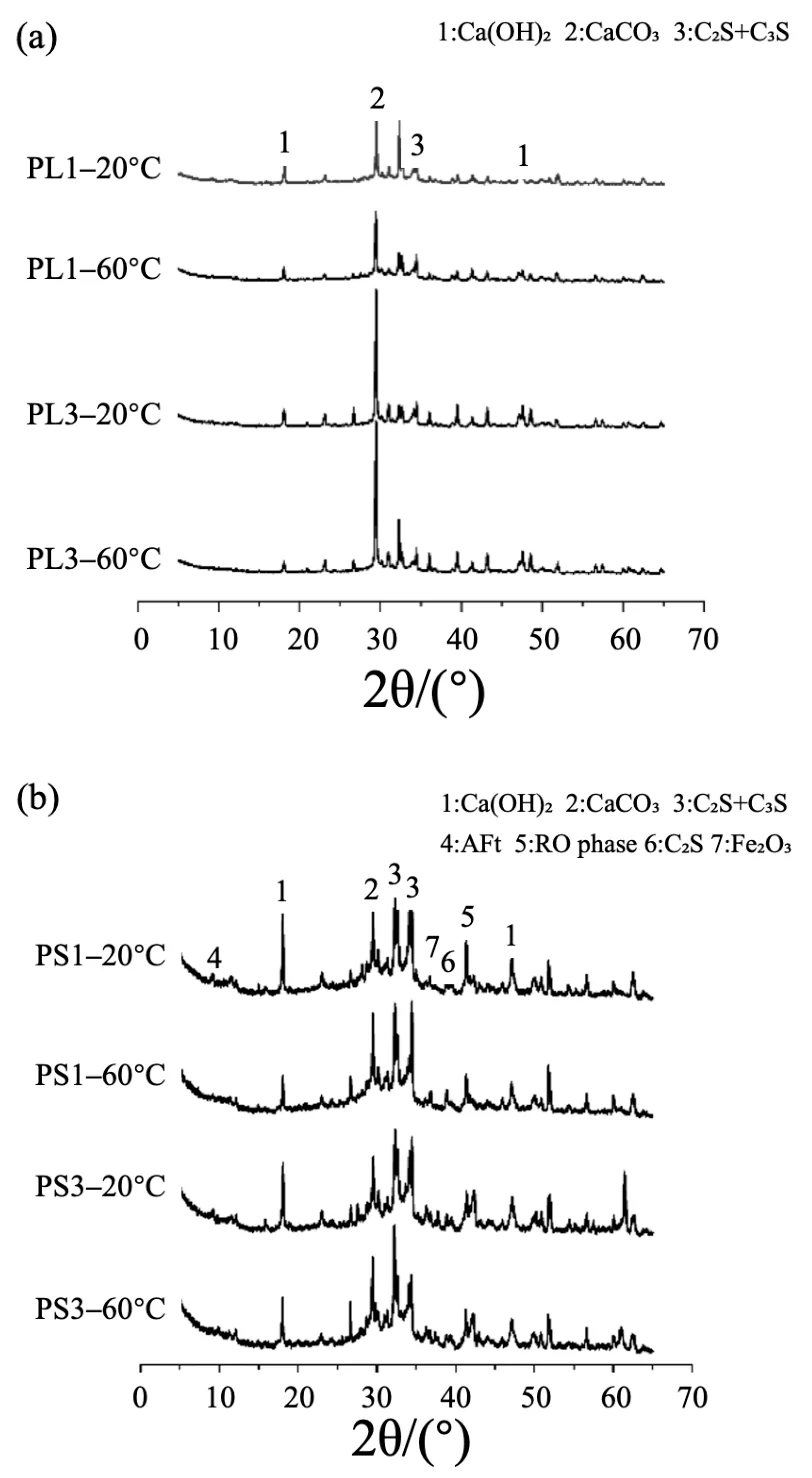
XRD patterns of the hardened paste at extremely low w/b ratio with steel slag powder or limestone powder cured for 28 d under different conditions. (**a**) SCC and (**b**) HTCC.

The XRD patterns of the hardened paste at an extremely low w/b ratio with limestone powder or steel slag powder cured for 28 d under different conditions are shown in Figure 6. Figure 6a shows that the diffraction peak intensities of C_3_S and C_2_S decrease with increasing limestone powder content under SCC. These findings indicate that an increase in limestone powder content promotes late-age hydration of the system. The changes in the diffraction peak intensities of CH with respect to the dosage of limestone powder under HTCC are similar to those under SCC. The content of highly active SCMs in sample HPL1 is high, and more CH is consumed in the pozzolanic reaction. As shown in Figure 6b, the diffraction peak intensities of CH and C3S + C2S decrease with increasing steel slag powder content under SCC, indicating that steel slag powder can promote later-age hydration of the system. The change rule of the diffraction peak intensities of CH and C_3_S + C_2_S with increasing steel slag powder content under HTCC is opposite to that under SCC. An increase in steel slag powder content decreases the content of GGBFS. The CH consumed by the pozzolanic reaction decreases accordingly. The hardened paste with steel slag powder has a stronger CaCO_3_ diffraction peak, which is more obvious under HTCC. A comparison of the data reveals that the diffraction peak intensity of the CH of the hardened paste with steel slag powder is stronger (Figure 6a,b). This is related to the higher degree of hydration and greater amount of hydration products. The change rules are similar under the two curing conditions.

### 3.4. Pore Structure

**Figure 7:**
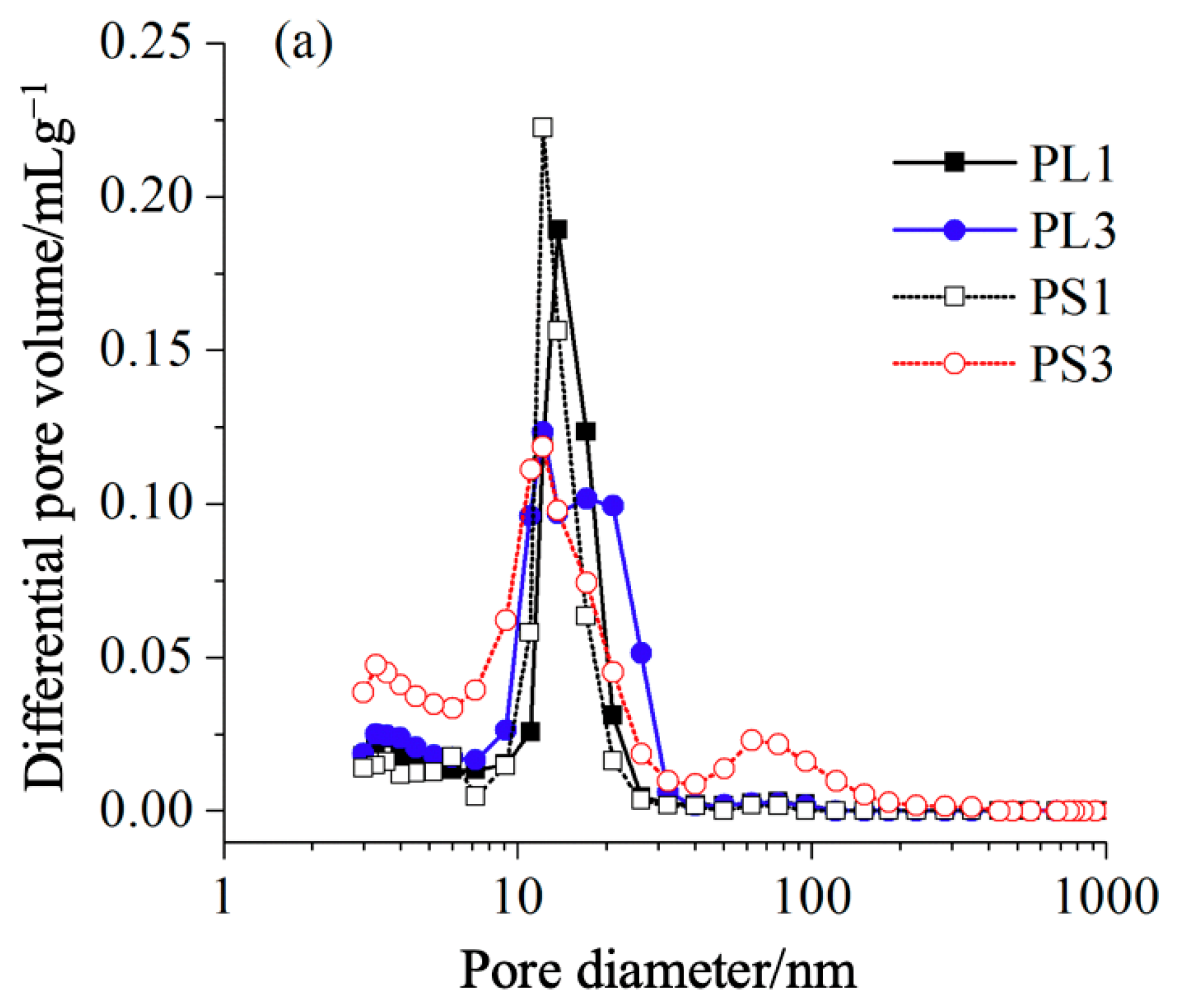

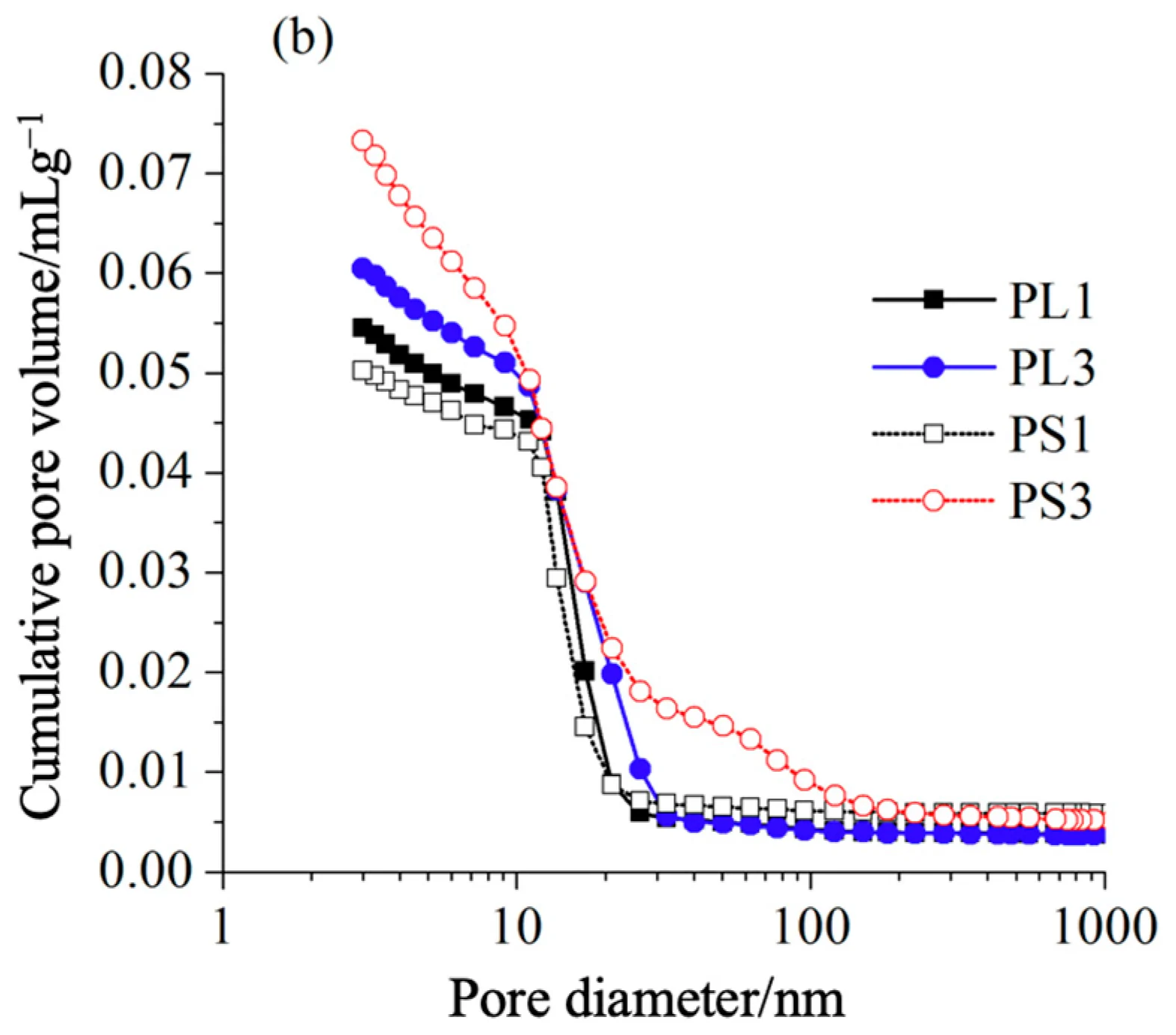
Pore structure of the hardened paste at extremely low w/b ratio with limestone powder or steel slag powder cured for 28 d under SCC. (**a**) Pore size distribution and (**b**) cumulative pore volume.

Figure 7 shows the differential pore volume and cumulative pore volume of hardened pastes at an extremely low w/b ratio with limestone powder or steel slag powder at 28 d under SCC. The peak is the critical pore diameter on the curve. Figure 7 shows that the critical pore diameter of the hardened paste with limestone powder first slightly decreases and then increases with increasing limestone powder content. There is no obvious change in the critical pore diameter of the hardened paste with increasing steel slag powder content, but the cumulative pore volume increases. A comparison between a sample with limestone powder and a sample with steel slag powder reveals that the critical pore diameter and cumulative pore volume of the hardened paste with limestone powder are greater than those of the hardened paste with steel slag powder when the content is 10%. These findings indicate that the refining effect of steel slag powder on the pore diameter is better than that of limestone powder when the dosage is low. This is related to the low reactivity of the limestone powder. When the dosage is 30%, the critical pore diameter of the hardened paste with limestone powder is greater. However, the cumulative pore volume of the hardened paste with steel slag powder is larger. According to the results of the Wn (Figure 4), the degree of reaction of the cementitious material with steel slag powder is greater at later age, but the filling effect of steel slag powder on the system is not as good as that of limestone powder, especially steel slag powder containing RO phase with a larger particle size (Figure 1). The content of pores larger than 30 nm in the sample with 30% steel slag powder is much higher (Figure 7b). These results indicate that the pore structure under SCC is controlled by hydration-product formation and physical filling. At a dosage of 10%, the later reaction of steel slag powder and GGBFS generate hydration products that fill or divide pores. However, at a dosage of 30%, the reduction in reactive GGBFS and the increase in coarse, slowly reacting steel slag particles become dominant, resulting in a larger pore volume. This interpretation is consistent with the chemically bound water results (Figure 4).

**Figure 8:**
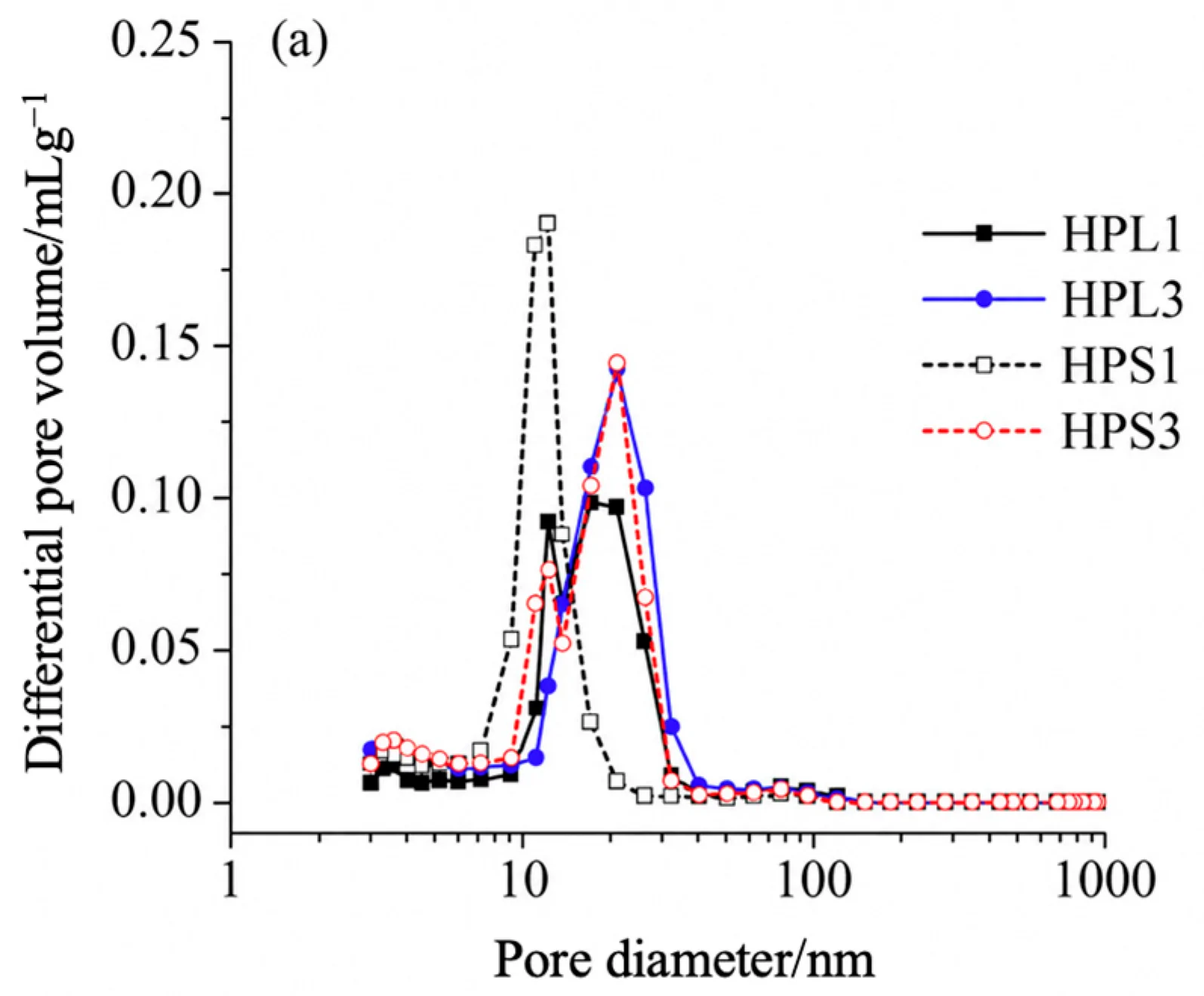

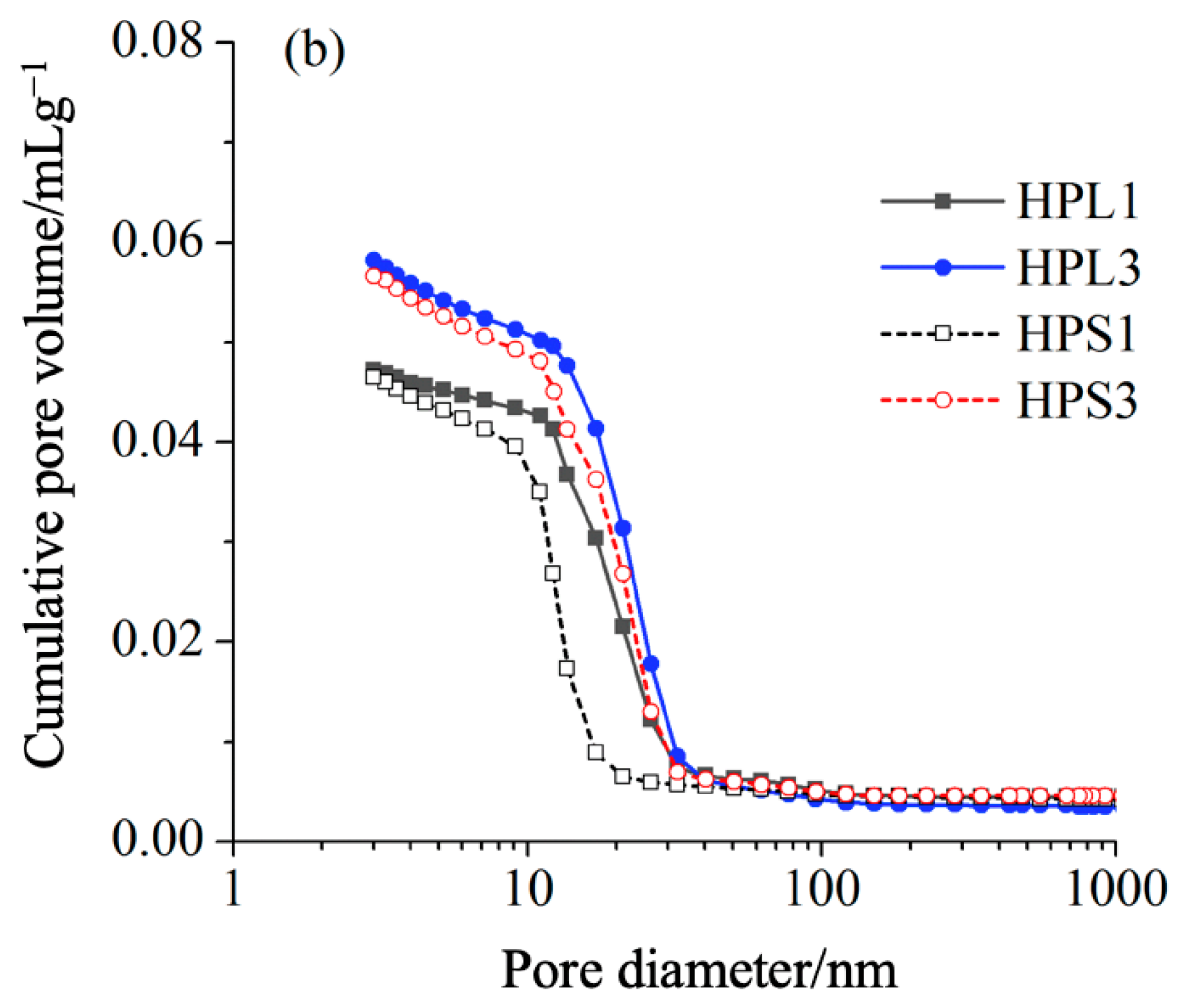
Pore structure of hardened paste at extremely low w/b ratio with limestone powder or steel slag powder cured for 28 d under early HTCC. (**a**) Pore size distribution and (**b**) cumulative pore volume.

Figure 8 shows the differential pore volume and cumulative pore volume of hardened paste at an extremely low w/b ratio with limestone powder or steel slag powder at 28 d under HTCC. The critical pore diameter and cumulative pore volume of the hardened paste increase with increasing limestone powder or steel slag powder content (Figure 8). This may be related to the reduction in GGBFS content. Compared with that of limestone powder, the pore-refining effect of steel slag powder is more obvious. This is also one of the reasons for the large difference in later-age compressive strength between concrete with steel slag powder and concrete with limestone powder at an extremely low w/b ratio (Figure 3).

**Figure 9:**
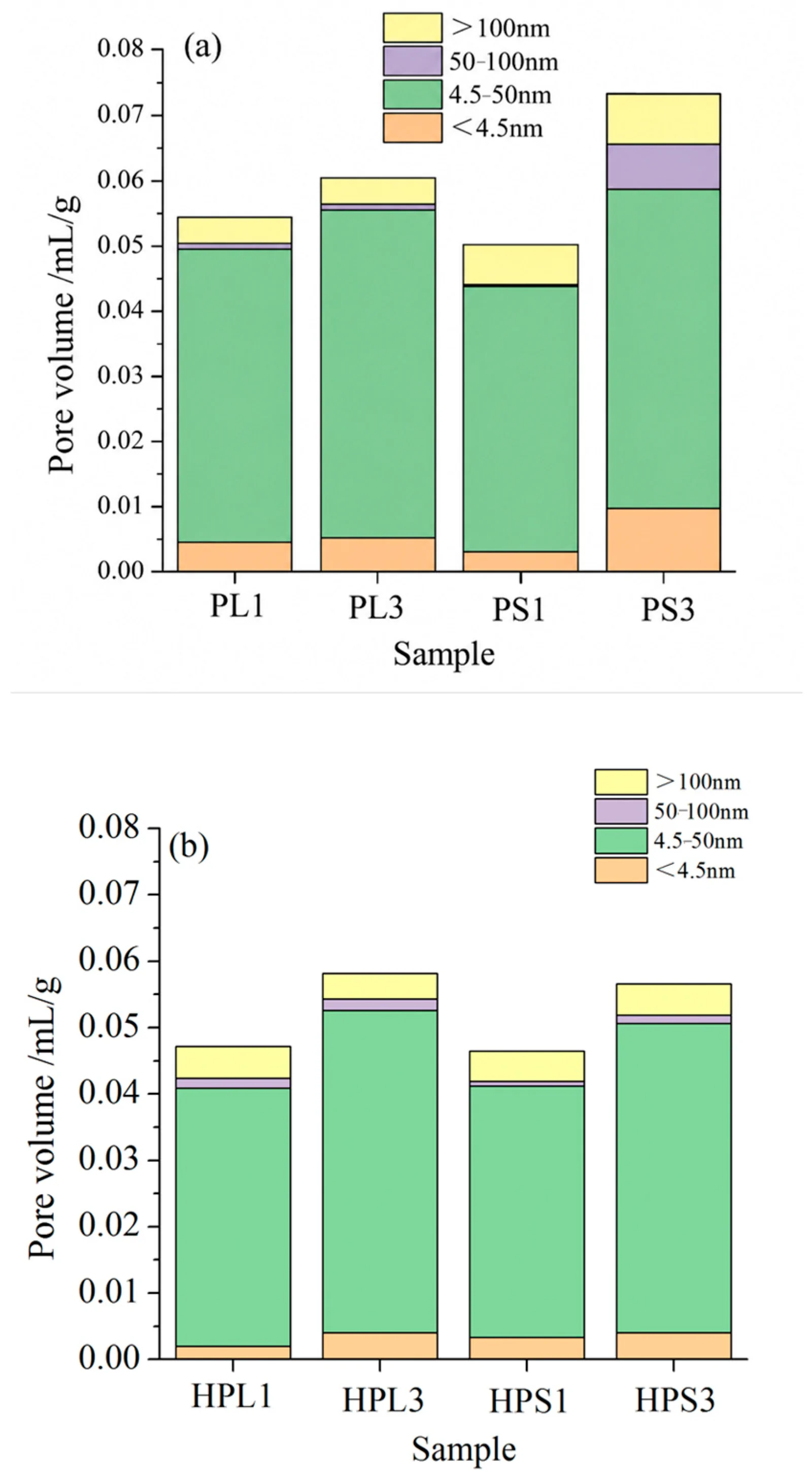
Pore volume of the hardened paste at extremely low w/b ratio with steel slag powder or limestone powder at 28 d. (**a**) SCC and (**b**) early HTCC.

Figure 9 shows the pore volume of the hardened paste at an extremely low w/b ratio with limestone powder or steel slag powder at 28 d under different curing conditions. There are several ways to classify the pore structure of paste. It can be divided into four types according to the pore size: <4.5 nm, 4.5–50 nm, 50–100 nm and >100 nm. Pores with diameters larger than 100 nm are multiple harmful pores, and their number and size dramatically affect the strength and permeability of concrete. As shown in Figure 9a, the cumulative pore volume generally increases with increasing limestone powder or steel slag powder content under SCC. This change rule is more obvious for the hardened paste with steel slag powder. The pore structure of paste with steel slag powder is better when the content is 10%, but the pore structure becomes worse when a large amount of steel slag powder content is added. The volume ratio of harmless pores (<4.5 nm) and less harmful pores (4.5–50 nm) in paste with limestone powder is larger. The physical effect of steel slag powder on the microstructure is not as good as that of limestone powder, which can better fill large pores between particles and reduce porosity. Figure 9b shows that the pore volume gradually increases with increasing limestone powder or steel slag powder content under HTCC. The porosity of paste with steel slag powder is lower and the volume proportion of harmless pores and less harmful pores is higher under early HTCC. The high temperature stimulates the activity of steel slag powder, and the continuous reaction of steel slag powder refines the pore size. A comparison of Figure 9a with Figure 9b reveals that when the dosage is 10% and 30%, the volume of multiple harmful pores in the hardened paste with limestone powder increases under early HTCC compared with that under SCC. When the dosage is 30%, the cumulative pore volume of the hardened paste with limestone powder slightly decreases under early HTCC. However, the total pore volume and the volume of multiple harmful pores in the hardened paste with steel slag powder dramatically decrease under early HTCC, which is consistent with relevant research results [17]. The pore structure of the hardened paste with limestone powder becomes loose, and the pore structure of the hardened paste with steel slag powder becomes denser under HTCC. The decrease in large harmful pores in the steel slag systems suggests that high temperature promoted hydration-product formation, thereby filling pores and refining the pore network. This pore refinement is consistent with the higher chemically bound water content, and improved compressive strength of the steel-slag-containing samples. In contrast, limestone powder mainly contributes through physical filling; at a high dosage, the reduced GGBFS content limits hydration-product formation and weakens the pore-refining effect.

### 3.5. Morphology

**Figure 10:**
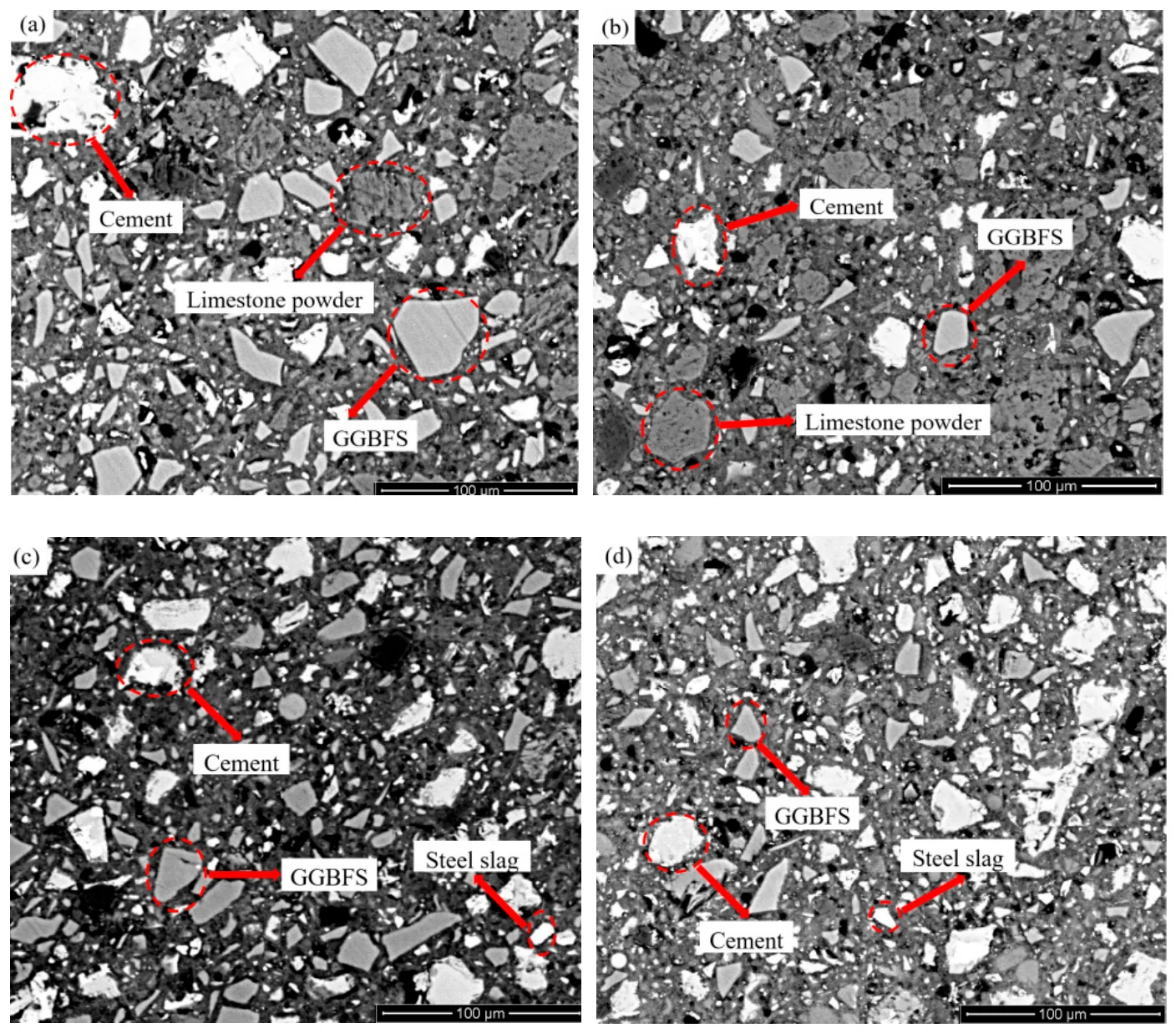
Morphology of hardened paste at extremely low w/b ratio with steel slag powder or limestone powder cured for 28 days under SCC. (**a**) PL1, (**b**) PL3, (**c**) PS1 and (**d**) PS3.

Figure 10 shows the BSE image of the hardened paste at an extremely low w/b ratio with limestone powder or steel slag powder at 28 d under SCC. A comparison of Figure 10a,b reveals that the number of unreacted cement particles decreases and that the number of pores gradually increases with increasing limestone powder content. The limestone powder is homogeneous gray and has no trace of etching. These findings indicate that the limestone powder hardly participates in the reaction and mainly plays a role in particle filling in the composite binder paste. Figure 10c,d show that the number of unreacted cement particles increases and that the number of pores gradually increases with increasing steel slag powder content. A comparison of the images of the hardened paste with limestone powder and the hardened paste with steel slag powder reveals that the number of unhydrated cement particles and unreacted GGBFS particles in the hardened paste with limestone powder is lower when the dosage is 30%. A large amount of steel slag powder is harmful to the hydration of cement and the reaction of GGBFS under SCC. The microstructure of the hardened paste with 30% steel slag powder is looser than that of the hardened paste with 30% limestone powder.

**Figure 11:**
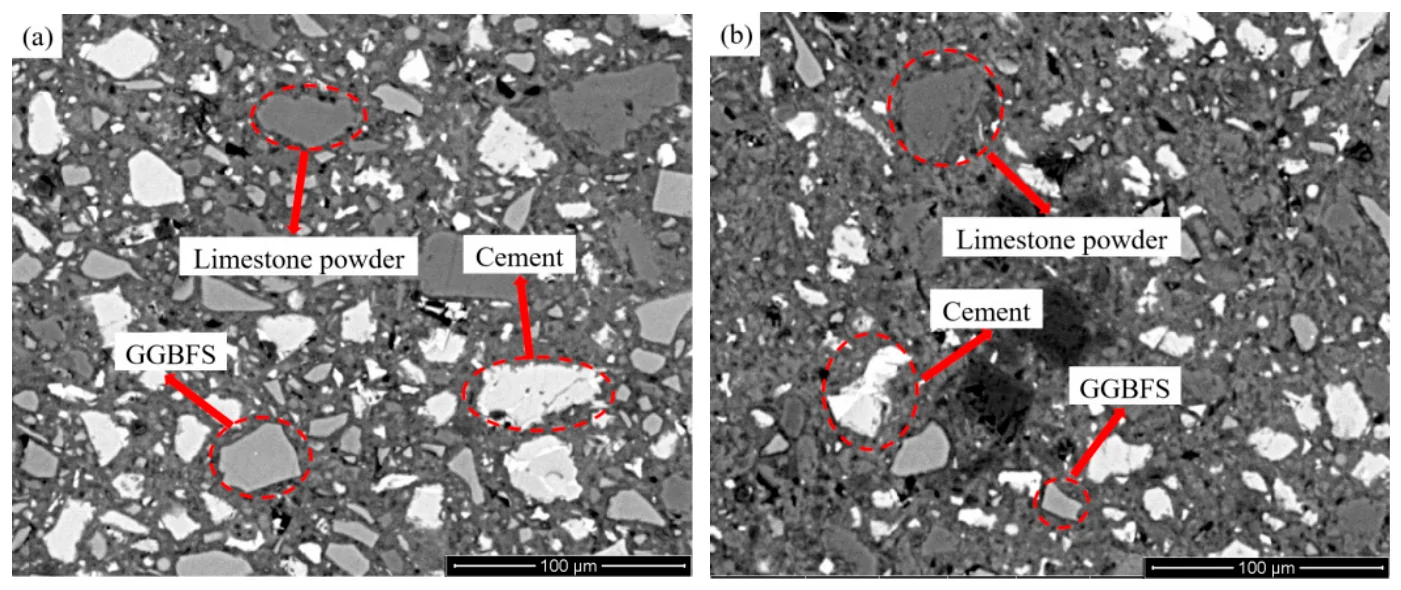

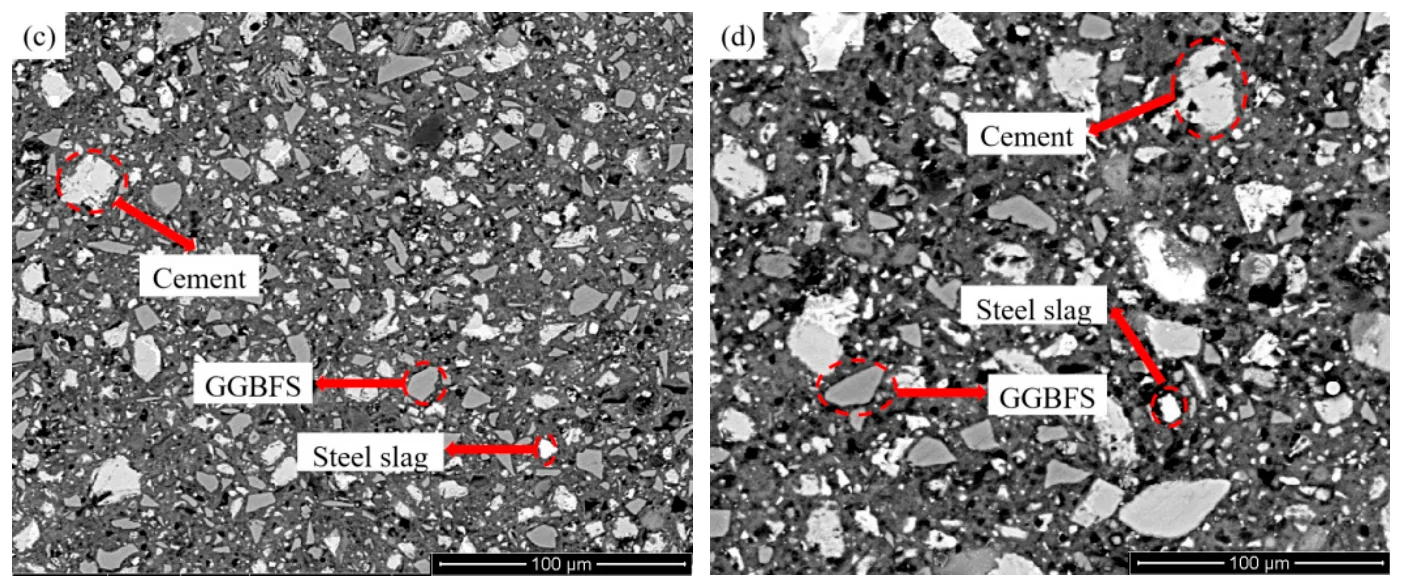
Morphology of hardened paste at extremely low w/b ratio with steel slag powder or limestone powder cured for 28 days under HTCC. (**a**) HPL1, (**b**) HPL3, (**c**) HPS1 and (**d**) HPS3.

Figure 11 shows the microscopic morphology of the hardened paste at an extremely low w/b ratio with limestone powder or steel slag powder at 28 d under early HTCC. A comparison of Figure 11a,b reveals that the microstructure of the hardened paste becomes looser and that the number of pores increases with increasing limestone powder content. This indicates that the reaction degree of the cementitious material is low and that the total amount of hydration products is small, which is consistent with the results of the Wn (Figure 5). The number of unhydrated cement particles significantly decreases and the number of pores reduces with increasing steel slag powder content (Figure 11c,d). The high temperature dramatically promotes the hydration of steel slag powder, which further promotes the reaction of GGBFS and the hydration of cement. A comparison of the BSE images of the hardened paste with limestone powder and the hardened paste with steel slag powder clearly reveals that fewer pores exist in the hardened paste with steel slag powder and that the structure is denser under early HTCC.

## 4. Conclusions

The effects of limestone powder or steel slag powder on the mechanical properties and microstructure of cement-based materials at an extremely low w/b ratio under different curing conditions were studied. On the basis of the analysis of compressive strength, chemically bound water, hydration products, pore structure and microscopic morphology, the mechanism of limestone powder and steel slag powder in cement-based materials at an extremely low w/b ratio was revealed, and the following conclusions were obtained:

(1) The early strength of concrete at an extremely low w/b ratio with limestone powder is slightly greater than that of concrete with the same amount of steel slag powder under SCC. The addition of steel slag powder has a better promoting effect on the later-age strength of concrete at an extremely low w/b ratio. The promotion effect of steel slag powder on the strength of concrete is more significant under HTCC. When the contents of limestone powder or steel slag powder are 10% and 20%, the compressive strength of concrete at an extremely low w/b ratio with limestone powder is greater. However, when the dosage reaches 30%, the later-age strength of concrete at an extremely low w/b ratio with steel slag powder is slightly greater than that of concrete with limestone powder.
(2) The early-age Wn of the hardened paste with limestone powder is high under SCC. However, the reaction rate of the cementitious material with steel slag powder is obviously faster at later ages. The Wn of the hardened paste with steel slag powder is high under HTCC.
(3) Under SCC, an increase in the limestone powder or steel slag powder content promotes late-age hydration of the system. The high temperature strongly promotes the pozzolanic reaction of the sample with steel slag powder.
(4) The volume of multiple harmful pores in the hardened paste with steel slag powder is smaller when the dosage is 10% under SCC, but the pore structure becomes worse when a large amount of steel slag powder content is added. The pore structure of the hardened paste with steel slag powder improves under HTCC.
(5) Under SCC, the microstructure of hardened paste with a relative large amount of limestone powder is denser than that of hardened paste with the same amount of steel slag powder. However, the opposite rule is found under HTCC.

Overall, the results support the feasibility of using 50% SCMs in an extremely low-w/b binder system oriented toward UHPC-level performance. Among the tested mixtures, 10–20% limestone powder is recommended for early-strength-oriented applications, whereas higher steel slag powder contents may be considered when HTCC and later-age strength development can be utilized. These recommendations are based on mechanical, hydration and microstructural results; workability, shrinkage and durability should be evaluated before practical application.

## Figures and Tables

**Table 1:** Chemical compositions of the raw materials (wt/%).

Composition	SiO_2_	Al_2_O_3_	Fe_2_O_3_	CaO	CaCO_3_	MgO	SO_3_	Na_2_O_eq_	f-CaO	Loss
Portland cement	21.18	4.73	3.41	62.49	-	2.53	2.83	0.56	0.72	1.76
GGBFS	34.55	14.36	0.45	33.94	-	11.16	1.95	0.63	-	0.70
Limestone powder	8.43	2.39	1.41	-	82.85	3.28	0.10	0.8	-	-
Steel slag powder	12.77	2.12	23.49	49.17	-	3.54	0.23	0.45	-	1.86
Silica fume	99	-	-	1	-	-	-	-	-	-

Na_2_O_eq_ = Na_2_O + 0.658K_2_O.

**Table 2:** Mix proportions of concrete/kg·m^−3^.

Sample	Cement	Silica Fume	GGBFS	Steel Slag Powder	Limestone Powder	Coarse Aggregate	Fine Aggregate	Water	HRWRA
CS1	425	85	255	85	0	1112	351	136	17
CS2	425	85	170	170	0	1112	351	136	17
CS3	425	85	85	255	0	1112	351	136	17
CL1	425	85	255	0	85	1112	351	136	17
CL2	425	85	170	0	170	1112	351	136	17
CL3	425	85	85	0	255	1112	351	136	17

**Table 3:** Mix proportions of paste/%.

Sample	Cement	Silica Fume	GGBFS	Steel Slag Powder	Limestone Powder	Water	HRWRA
PS1	50	10	30	10	0	16	2
PS2	50	10	20	20	0	16	2
PS3	50	10	10	30	0	16	2
PL1	50	10	30	0	10	16	2
PL2	50	10	20	0	20	16	2
PL3	50	10	10	0	30	16	2

## Data Availability

The original contributions presented in this study are included in the article. Further inquiries can be directed to the corresponding author.
